# Biomarkers and Gene Polymorphisms in Members of Long- and Short-lived Families: A Longevity Study

**DOI:** 10.2174/1874192401812010059

**Published:** 2018-07-31

**Authors:** Vana Kolovou, Olga Diakoumakou, Athanasia K Papazafiropoulou, Niki Katsiki, Elisabeth Fragopoulou, Ioannis Vasiliadis, Dimitris Degiannis, Leonidas Duntas, Smaragdi Antonopoulou, Genovefa Kolovou

**Affiliations:** 1 Molecular Immunology Laboratory, Onassis Cardiac Surgery Center, Athens, Greece; 2 Department of Science Nutrition-Dietetics, Harokopio University, Athens, Greece; 3 Cardiology Department, Onassis Cardiac Surgery Center, Athens, Greece; 4 1^st^ Department of Internal Medicine and Diabetes Center, Tzaneio General Hospital of Piraeus, Piraeus, Greece; 5 2^nd^ Propedeutic Department of Internal Medicine, Hippokration University Hospital, Thessaloniki, Greece; 6 Evgenideion Hospital, Unit of Endocrinology Diabetes and Metabolism, University of Athens, Athens, Greece

**Keywords:** Uric acid, Adiponectin, Insulin-like growth factor, Cholesteryl ester transfer protein, Angiotensin-converting enzyme, Insulin-like growth factor binding protein-3 genes, Lifespan, Longevity

## Abstract

**Background:**

The influence of biomarkers in human lifespan has been investigated but with no clear results yet.

**Materials and methods:**

Lipids, Uric Acid (UA), Adiponectin (ADIPOQ), Insulin-like Growth Factor (IGF-1), cholesteryl ester transfer protein (CETP) and angiotensin-converting enzyme (ACE) proteins, as well as *CETP*, *ADIPOQ*, *insulin-like growth factor binding protein-3* (*IGFBP3*) and *ACE*-gene polymorphisms were evaluated in 149 Greek individuals. The Long-Lived Families (LON) (n=84) comprised of 3 generations: long-lived aged ≥90 years (P), offspring (FL1) and their grandchildren (FL2), while the Short-Lived Families (EAD) (n=65) where both parents died <75 years, comprised of 2 generations: middle-aged (FD1) and children (FD2).

**Results:**

Serum CETP and IGF-1 levels were lower, whereas AdipoQ concentrations were higher in P compared with FL1 and FL2 members (CETP: p = 0.03 for both comparisons; IGF-1 p < 0.001 for both comparisons and ADIPOQ: p = 0.001 and p = 0.004, respectively). Furthermore, serum triglycerides, UA and glucose concentrations were higher in FD1 compared with FD2 subjects (p=0.001, 0.02 and ≤0.001, respectively). In FD2 and FL2, CETP levels were lower in individuals with *B2B2* compared with *B1B1* genotype (p=0.007). Additionally, ACE concentrations were higher in individuals with DD compared with *II* genotype in both Families (p=0.001). After adjustment for age and gender, CETP levels were lower in P and FL2 individuals with *B2B2* compared with the *B1B1* genotype (p=0.004 and 0.007, respectively).

**Conclusion:**

Increase serum TGs, UA and GL concentrations were higher in the middle-aged individuals compared with their children in families independently of their lifespan. The serum adiponectin concentration was the highest in the oldest old individuals implying beneficial influence on lifespan. Independently of family’s lifespan history, the youngest individuals with *CETPB2B2* genotype, compared with individuals with *CETPB1B1* genotypes, had lower serum CETP concentrations. The knowledge of the unfavourable gene(s)influencing human lifespan may be helpful in encouraging individuals to follow healthier lifestyle habits and better control their high-risk biomarkers.

## 1 INTRODUCTION

Up-to-date, genetic studies have identified a limited number of loci associated with human longevity by recognizing age at death or survival up to advanced ages as a specific phenotype. Long-lived people are those who have exceeded ≥90 years [1], some of them having overcome or stabilized or avoided deadly diseases such as cancer and atherosclerosis. The abovementioned characteristics of long-lived populations which led to their longer lifespan imply that beyond environmental factors, genes controlling lifespan may play an important role [2].

Atherosclerosis is a disease that begins very early in life, even in the prenatal and infancy period, and becomes clinically evident in approximately the 4th or 5th decade of life [3]. The diseases sharing the atherosclerotic mechanism are mainly Coronary Artery Disease (CAD), Diabetes Mellitus (DM), hypertension, Alzheimer’s Disease (AD) and obesity [4]. Several genes have been linked to the risk of the progression of these diseases, such as cholesteryl ester transfer protein (*CETP*) [5], Apolipoprotein E (*ApoE*) [6, 7], Angiotensin-Converting Enzyme (*ACE*) [8] and adiponectin (*ADIPOQ*) [9]. Genes that may be involved in cancer pathogenesis include Insulin-like Growth Factor (*IGF*), *p53* and fork-head box O3A (*FOXOA3*) [10]. In the present study, we focused on 5 Single Nucleotide Polymorphisms (SNPs) of the *CETP*, *ADIPOQ*, *IGFBP3* and *ACE* genes and their related proteins.

CETP interacts with lipoproteins in order to exchange cholesterol esters between Triglycerides (TGs), Low-Density Lipoproteins (LDL) and High-Density Lipoproteins (HDL). High levels of CETP may enhance the formation of small, dense LDL and HDL particles, which are atherogenic [11]. Several *CETP* gene variants such as *TaqIB* (rs708272) and *I405V* (rs5882) are associated with reduced CETP mass and HDL cholesterol (HDL-C) [12, 13]. Both *TaqIB* and *I405V* variants are the most widely studied and were found to be associated with CAD [5, 14, 15], left main CAD [16] and longevity [17, 18].

The renin-angiotensin-aldosterone system (RAAS) also plays a role in the maintenance of cardiovascular (CV) homeostasis. The *ACE* is an important gene of the RAAS that has been evaluated in the pathogenesis of hypertension, CAD, heart failure and, recently, longevity [19]. The most common variant of *ACE* gene, *rs1799752,* is associated with hypertension [20], heart failure [21] and lifespan variation [19].

The adipose tissue-derived peptide, AdipoQ, is a cytokine [22]. AdipoQ is a determinant of insulin sensitivity that exerts anti-inflammatory and anti-atherogenic effects [22]. The endocrine function of adipose tissue seems to contribute to several metabolic disorders as well as CV Disease (CVD) [23-25]. Human adipocytes appear to have a number of receptors that are sensitive to various factors that influence important systems such as the endocrine, vascular, immune and nervous system [26]. A common variant of the *ADIPOQ* gene in chromosome 3 and exon 2 and position +45T>G with *rs2241766* (+45T>G) was associated with the risk of CVD [27].

The *IGF-1* gene seems to be negatively related to age [28]. Circulating IGF binding protein-3 (IGFBP3) binds > 90% of the circulating IGF-I, thereby reducing the levels of free IGF-I and increasing the risk of DM [29]. Furthermore, the IGFBP3 exerts mitogenic and metabolic activities in growth regulation, survival and cell differentiation [30]. The *rs2854744* (*A-202C*) variant of the *IGFBP3* gene was found to be associated with circulating IGFBP-3 levels [31]. There is some evidence that the alleles related to higher circulating IGFBP-3 levels are also associated with a higher risk of early-stage cancers [32].

In the present study, we evaluated serum levels of specific proteins as well as variants of their related genes in long-lived families comprising 3 generations and short-lived families comprising 2 generations. It is suggested that some people may have protective genes and proteins in contrast to people with a disease history “cargo”. This is the first time that potential longevity genes were studied in comparison with their serum protein levels in Greek families. A unique characteristic of this study was that samples were collected from all generations of both family groups, even from the oldest old members. The aim of this study was to investigate the differences in gene variants and serum protein levels between the members of long- and short-lived families.

## 2 SUBJECTS AND METHODS

### 2.1 Sample Collection

This study was designed and performed in agreement with the recommendations for the human genotype-phenotype association studies published by the National Cancer Institute-National Human Genome Research Institute (NCI-NHGRI) Working Group on Replication in Association Studies [33] indicating time and location of subject recruitment, success rate for DNA acquisition, internal control samples (from the same DNA) and sample tracking methods.

The study protocol was approved by the institutional ethics committee (Onassis Cardiac Surgery Center, Athens, Greece) and the Harokopio University (Athens, Greece) and was in accordance with the Declaration of Helsinki for Human Research of 1974 (last modified in 2000). All participants were of Caucasian origin and descent for ≥3 generations.

The Longevity Group (LON) consisted of the oldest old aged ≥90 years (P), one of their children (FL1) and one of their grandchildren (FL2). Families whose both parents died <75 years of any aged-related disease and had no history of individuals living >90 years [Early Death Group (EAD)] consisted of middle-aged individuals (FD1) and one of their children (FD2).

### 2.2 Genotyping

Genotyping was performed specifically for research purposes. Extraction of genomic DNA was performed from leukocytes separated from whole blood using a standard method with FlexiGene^®^ DNA kit (Qiagen, Venlo, Netherlands).

The study variants were detected using Polymerase Chain Reaction (PCR) and Restricted Fragment Length Polymorphism analysis (RFLP) (details are included in the Supplementary Data).

RFLP results were validated in the following way: 1) around 20% of all samples were repeated to confirm findings of the PCR-RFLP method, and, 2) randomly selected PCR-RFLP results were confirmed by direct automated sequencing of PCR products for each polymorphism using the BigDye terminator chemistry kit (ABI, USA) and the 3,500 genetic analyser (ABI, USA). The concordance between repeated samples, sequencing and our results was 100%.

The 5 gene polymorphisms which were evaluated were: *CETP TaqIB* (rs708272, genotypes: *B1B1, B1B2, B2B2*), *CETP I405V* (rs5882, genotypes: *II, IV, VV*), ACE (rs1799752, genotypes: *II, ID, DD*), ADIPOQ (rs2241766, genotypes: *GG, GT, TT*) and IGFBP3 (rs2854744, genotypes: *AA, AC, CC*).

### 2.3 ELISA Measurements

Up to 4 ml of blood samples were obtained in BD (Becton Dickinson Diagnostics, NJ, USA) vacutainers. The blood was stored at room temperature for 45 min, after which the serum was separated by centrifugation at 1500xg, divided into aliquots, snap frozen and stored at -80°C until assay. The serum levels of each component were measured using commercially available Quantikine human ELISA kits: R&D systems, Minneapolis, MN, USA for AdipoQ, IGF-1, ACE and ALPCO Diagnostics, Salem, NH for CETP. The tests were performed according to the manufacturer’s specifications for each ELISA kit. The sensitivity of the assay for AdipoQ was 0.246 ng/mL, for IGF-1 0.026 ng/mL, for ACE 0.019 ng/mL and for CETP 0.2 μg/mL. The assay range for AdipoQ was 0.9-21.4 μg/mL, for IGF-1 40-258 ng/mL, for ACE 37.2-202 ng/mL and for CETP 0.2-5.0 μg/mL.

### 2.4 Lipid Profile, Uric Acid (UA) and Glucose (GL)

Serum Total Cholesterol (TC), TGs, HDL-C, GL and UA were measured using enzymatic colorimetric methods using a photometric analyzer (PowerWave X52, BioTek Instruments, Potton, UK) with commercially available kits (Biosis, Biotechnological Applications, Athens, Greece). The serum LDL cholesterol (LDL-C) concentration was calculated using the Friedewald formula only in subjects with TGs concentration <400 mg/dl.

Body Mass Index (BMI) was calculated according to the following formula: weight (kg)/ [height (m)]^2^. In the P group, usually, the weight (kg) and height (m) were measured with difficulty due to posture problems. This was carried out at the patient’s home by a doctor who was taking the blood samples. In all other groups the weight and height were measured by a nurse in Outpatient Clinics.

### 2.5 Statistical Analysis

The normality of continuous variables was tested using the Shapiro-Wilk test. Levels of the quantitative variables are presented as median (25**-**75 percentile). Comparisons of mean values of age, BMI, TC, TGs, HDL-C, LDL-C UA, GL, CETP, ACE, IGF-1 and ADIPOQ between study groups (FD1-FD2 in the EAD Group, P-FL1, FL1-FL2, P-FL2 in the LON Group, and FL1-FD1, FL2-FD2 for LON *vs* EAD) were performed with the student unpaired t test. Categorical variables were compared with the use of the chi-square and Fisher’s exact tests. In order to assess the association of serum CETP, ACE, IGF1 and AdipoQ levels with the examined genotypes, multivariate regression analysis of gene polymorphisms and their related proteins, after adjustment for age and gender, was performed (p values of 0.05 were considered significant). The Bonferroni correction was used for the comparison (by one-way ANOVA) of mean values of TC, TGs, HDL-C, LDL-C, UA, GL, CETP, ACE, IGF-1 and ADIPOQ between EAD *vs* LON groups. Therefore, p value based on the Bonferroni correction is a=0.05/2 (number of statistical tests between 2 groups) x 10 (number of factors of interest) =0.0025.

Data were analyzed using the statistical software package SPSS 19.0 (SPSS Inc, Chicago, IL, USA).

## 3 RESULTS

### 3.1 Demographic Data of the LON Group (Table **1**)

The BMI among members of the LON Families should be interpreted with caution, since the height in P individuals was difficult to measure accurately. Serum TGs were lower in FL2 members compared with P and FL1, whereas serum HDL-C was higher in FL2 compared with FL1 members (p = 0.001 and p = 0.002, respectively). A trend towards higher serum UA levels in P compared with FL2 individuals was found (p = 0.06). Serum GL levels were higher in P compared with FL2 and in FL1 compared with FL2 (p = 0.002 and p = 0.04, respectively). Serum CETP and IGF-1 levels were lower, whereas AdipoQ concentrations were higher in P compared with FL1 and FL2 members (CETP: p = 0.03 for both comparisons; IGF-1 p < 0.001 for both comparisons and ADIPOQ: p = 0.001 and p = 0.004, respectively).

### 3.2 Demographic Data of the EAD Group (Table **1**)

The FD1 members were older and had a higher BMI compared with FD2 (p < 0.001). Serum TGs, UA and GL levels were higher, whereas IGF-1 concentrations were lower in FD1 members compared with FD2 (TG: p = 0.001, UA: p = 0.02, GL p < 0.001, IGF-1: p < 0.001, respectively).

The comparison of serum CETP, IGF-1 and AdipoQ concentrations between parents and their offspring was not performed, because by definition the parents were not alive.

### 3.3 Comparison of Demographic and Clinical Data between the LON and EAD Groups

The FL1 members were 5 years older than FD1 (p < 0.001) (Table **1**).

### 3.4 Relationships of Genotypes According to Encoding Protein

In the FD2 individuals of the EAD Group, serum CETP levels were lower for those with the *B2B2* genotype (FD2: b = - 0.36, p = 0.05) as well as in the LON Group (FL2: b = - 0.45, p = 0.03), in comparison to the *B1B1* genotype (Table **2a**).

In the EAD Group, ACE levels were higher for those with the *DD* genotype (FD1: b = 23.28, p = 0.009; FD2: b = 21.96, p = 0.007) as well as in the LON Group (FL1: b = 41.33, p = 0.02; FL2: b = 25.11, p < 0.001), in comparison to the *II* genotype (Table **2a**).

### 3.5 Multivariate Regression Analysis

Multivariate regression analysis of gene polymorphisms and their related proteins after adjustment for age and gender showed lower serum CETP levels in P and FL2 individuals with the *B2B2* genotype compared with individuals with the *B1B1* genotype (p = 0.004 and p = 0.007, respectively). Furthermore, ACE concentrations were higher in individuals with the *DD* genotype compared with the *II* genotype in both groups (LON: p = 0.001 and EAD: p = 0.03, Table **2b**). Mean values of TC, TGs, HDL-C, LDL-C, UA, GL, CETP, ACE, IGF-1 and ADIPOQ did not differ significantly between EAD vs LON groups.

## 4 DISCUSSION

In the present study, in line with a previous one [34], we compared biochemical and genetic markers between members of families with different lifespans.

The FL1 participants were 5 years older than FD1 group. There is no explanation for this and the difference is too small to have any influence on evaluated proteins levels as well as variants of their related genes.

Higher levels of UA were detected in FD1 individuals compared with FD2. UA as the last product of purine metabolism is a biomarker that is related to inflammation [35]. It is suggested that UA can penetrate cell membrane and exert damaging intracellular actions such as oxidation and inflammation [36]. This action could represent a link between UA and CAD. Apart from CAD, elevated UA may play an important role in the onset of hypertension and diabetic complications [37-40]. In the same line, Malik *et al.* [41] who studied healthy octogenarians determined that higher levels of UA were associated with vascular inflammation. In addition, Yan *et al.* [42] reported that elevated levels of UA were associated with an increased risk of cancer incidence and mortality, while Beavers *et al.* [43] suggested that elevations in UA might lead to sarcopenia. Accumulating evidence suggests that hyperuricemia is one of the important factors that may significantly contribute to the development and progression of CVD and chronic kidney disease [44]. Therefore, it follows that in members of families with a history of shorter lifespan, UA levels were similar between the middle-aged [FD1: 58.5 (51.7-66.3) years] and the oldest old individuals of long-lived families [93 (90-96) years]. Although it is premature to draw any definitive conclusions due to the small samples, these findings may suggest that in families with shorter lifespan, the increase in UA could be an additional factor involved in shortening lifespan.

Additionally, serum GL levels were increased in both groups according to age. The relationship between age, CVD and GL levels [45, 46], as well as the association between increased TGs and CVD [11, 47-49] have been previously investigated. We detected the highest TG levels in the FD1 individuals of the EAD Group. This may imply that while they are ageing, TG concentrations increase in members of short-lived families and this may be an additional CVD risk factor in middle-aged individuals of such families.

In the LON Group, HDL-C was not significantly different in the FL2 compared with the FL1 group (the difference was only 1 mg/dl). In contrast, HDL-C was significantly higher in the FL2 compared with the P group, but the number of samples was small.

AdipoQ levels were increased in the oldest individuals of the LON Group, which may imply that higher adiponectin levels act beneficially in very old individuals. Several studies reported higher adiponectin concentrations in centenarians compared with younger individuals [50-52]. Substantiating this finding, Bik *et al.* [53] described hyperadiponectinemia in centenarians and found an inverse correlation between AdipoQ and Homeostasis Model Assessment for Insulin Resistance (HOMA-IR).

Independently of family’s lifespan history, the FD2 and FL2 individuals with *CETPB2B2* genotype, compared with individuals with *CETPB1B1* genotypes, had lower serum CETP levels (p = 0.05, p = 0.03, respectively). However, after adjustment for age and gender, only individuals from the LON group, and more specifically, P and FL2 individuals, had this association. This suggests the possibility of genetic determination in these families and that the *B1* allele is unfavourable for long life. Furthermore, low CETP concentrations may act protectively in conjunction with the *B2* allele.

It is well-established that the *B2* homozygotes have less serum CETP activity or mass than *B1* homozygotes (see review by Boekholdt and Thompson [12]). However, we found this correlation only in the families with longevity.

In the present study, Greek individuals of both families with the *DD* genotype had higher serum ACE levels compared with the *II* genotype of ACE I/D polymorphism. Yang *et al.* [54] reported that in Taiwanese individuals with AD, the *DD* genotype was related to increased levels of ACE in the plasma and that the *I* allele was associated with a decreased risk of AD; they thus characterized the D allele as a “risk” allele. Several studies linked the increased ACE concentrations with the *DD* genotype, CAD [55], hypertension [56], autoimmune diseases [57, 58] and acute respiratory distress syndrome [59]. Moreover, Zhang *et al.* [60] reported that the *DD* genotype was more frequent in patients with major adverse CV events among CAD patients. On the other hand, there are studies that did not find any association of *ACE I/D* polymorphism and serum ACE levels in septic patients [61], age-related muscular degeneration [62] and patients with venous thromboembolism [63]. Multivariate regression analysis of gene polymorphisms and their related proteins after adjustment for age and gender also showed that ACE concentrations were higher in individuals with the *DD* genotype compared with the *II* genotype in both families.

Human lifespan depends mainly on 2 factors: 1) expression of genes and epigenetics; both are responsible for plasma proteins, enzymes and molecule levels, and, 2) environmental effects, which can influence these variables. Therefore, we evaluated families with longevity or early deaths, so that all members of the same family had potentially similar lifestyle behaviour. The family environment has a critical role in the development of cardiometabolic disorders (such as smoking behaviour, eating habits, obesity and hypertension) in offspring and their children [64]. Additionally, the association of support from family for adoption of healthy eating habits and performing exercise with improvements of self-leadership (defined as a process of behavioural and cognitive self-evaluation and self-influence, in which an individual achieves the self-direction and self-motivation needed to make positive changes in behaviours) is also important as shown in cancer patients [65]. Thus, the parental lifespan history, biochemical phenotype and certain genes could be used as a practical approach for the early preventive measures and identification of families at risk for early death.

The main limitation of this study is the relatively small sample size. However, long-lived individuals represent a very selective group and it is therefore difficult to collect samples from these families. In the present study, samples were collected from all generations of both Family Groups, even from the oldest old members. Furthermore, it is the first time that Greek families both with a history of early death and with long lifespan were evaluated for potential longevity genes and biomarkers.

## CONCLUSION

Increase serum TGs, UA and GL concentrations were higher in the middle-aged individuals compared with their children in families independently of their lifespan. Serum adiponectin concentration was the highest in the oldest old individuals implying beneficial influence on lifespan. Independently of family’s lifespan history, the youngest individuals with *CETPB2B2* genotype, compared with individuals with *CETPB1B1* genotypes, had lower serum CETP concentrations. The knowledge of the unfavourable gene(s) influencing human lifespan may be helpful in encouraging individuals to follow healthier lifestyle habits and better control their high-risk biomarkers.

## Figures and Tables

**Table 1:** Comparison of biochemical data between the Longevity and the Early Death Group.

–		**Early Death (EAD) Group**			**p**	**Longevity (LON) Group**			**p**	**p** **LON *vs* EAD**
–		**FD1 (n=34)**	**FD2** **(n=31)**		**(FD1-FD2)**	**P (n=28)**	**FL1 (n=28)**	**FL2 (n=28)**	**P-FL1,** **FL1-FL2,** **P-FL2**	**(FL1-FD1,** **FL2-FD2)**
**Age (years)**		58.5 (51.7-66.3)	33.0 (26-38)		**<0.001**	93 (90-96)	63.5 (58.3-66.7)	31.0 (26.3-39.0)	**<0.001** **<0.001** **<0.001**	**0.02** 0.72
**Gender**	**M**	16 (47) 18 (53)	15 (48) 16 (52)	0.79		11 (39) 17 (61)	11 (39) 17 (61)	14 (50) 14 (50)	1.00 0.42 0.42	0.54
	**F**									0.90
**BMI (kg/m^2^)**		27 (25-30)	23 (21-26)		**0.005**	23 (22-27)	27 (24-30)	23 (21-26)	**0.009** **0.003** 0.62	0.96 0.58
**TC (mg/dL)**		195 (160-238)	183 (135-209)		0.07	185 (144-214)	192 (149-226)	173 (155-206)	0.36 0.19 0.78	0.56 0.81
**TGs (mg/dL)**		101 (72-151)	53 (39-72)		**0.001**	77 (62-129)	75 (54-130)	44 (31-68)	0.70 **0.002** **0.001**	0.23 0.68
**HDL-cholesterol (mg/dL)**		51 (33-58)	51 (41-61)		0.23	44 (35-52)	45 (36-71)	55 (50-61)	0.28 0.28 **0.02**	0.30 0.21
**LDL- cholesterol (mg/dL)**		119 (87-151)	111 (72-146)		0.21	116 (85-1150)	119 (81-154)	101 (94-124)	0.74 0.34 0.51	0.59 0.92
**UA (mg/dL)**		4.6 (3-6)	4.1 (3-5)		**0.02**	4.7 (4-6)	4.4 (3-6)	4.1 (3-5)	0.23 0.48 0.06	0.28 0.62
**GL (mg/dL)**		101 (97-114)	90 (83-96)		**<0.001**	103 (89-123)	98 (88-113)	88 (82-97)	0.44 **0.04** **0.002**	0.74 0.55
**CETP** **(μg/mL)**		1.9 (1.7-2.4)	1.7 (1.5-2.3)		0.78	1.97 (1.7-2.3)	2.2 (1.9-2.5)	2.2 (1.9-2.7)	**0.03** 0.93 **0.03**	0.22 0.13
**ACE (ng/mL)**		145 (105-168)	126 (110-154)		0.32	140 (113-158)	157 (131-169)	136 (127-170)	0.14 0.55 0.26	0.29 0.10
**IGF-1 (ng/mL)**		72 (65-93)	123 (92-188)		**<0.001**	57 (36-74)	83 (67-107)	123 (101-160)	**<0.001** **<0.001** **<0.001**	0.19 0.82
**ADIPOQ (μg/mL)**		6 (3-9)	6 (4-11)		0.65	15 (9-21)	7 (4-11)	8 (6-133)	**0.001** 0.69 **0.004**	0.40 0.12
**Statin use, yes**		21 (62)	2 (6)		**<0.001**	9 (32)	12 (43)	0 (0)	0.40 - -	0.18 -

LON: Longevity, EAD: Early Death, P: older individuals, FL1: 1^st^ generation of longevity group, FL2: 2^nd^ generation of longevity group, FD1: 1^st^ generation of EAD Group, FD2: 2^nd^ generation of EAD Group. M: Male, F: Female, BMI: Body mass index, HDL: High density lipoprotein, LDL: Low density lipoprotein, UA: Uric acid, GL: Glucose, CETP: Cholesterol ester transfer protein, ACE: Angiotensin converting enzyme, IGF-1: Insulin-like growth factor-1. ADIPOQ: Adiponectin. Data are presented as median (25-75 percentile) or n (%). Bold means statistical significance with p < 0.05.

*The Bonferroni correction was used for the comparison (by one-way ANOVA) of TC, TGs, HDL-C, LDL-C, UA, GL, CETP, ACE, IGF-1 and ADIPOQ between EAD *vs* LON groups; p = 0.0025

**Table 2a:** Associations between gene polymorphisms and their related proteins.

–	**Early Death (EAD) Group**				**Longevity (LON) Group**					
–	**FD1**		**FD2**		**P**		**FL1**		**FL2**	
–	**b-value**	**p**	**b-value**	**p**	**b-value**	**p**	**b-value**	**p**	**b-value**	**p**
**CETP levels- *CETP* TaqIB genotype** **(B1B1 *vs* B2B2)**	-0.22	0.28	-0.36	**0.05**	-0.16	0.45	-0.021	0.89	-0.45	**0.03**
**CETP levels- *CETP* I405V genotype** **(II *vs* VV)**	-0.22	0.51	-0.31	0.26	-0.012	0.95	-0.046	0.87	-0.26	0.36
**ACE levels- *ACE* I/D genotype** **(II *vs* DD)**	23.28	**0.009**	21.96	**0.007**	6.45	0.44	41.33	**0.02**	25.11	**<0.001**
**IGF1levels- *IGFBP3* genotype** **(AA *vs* CC)**	-5.37	0.18	-0.011	0.99	0.89	0.87	7.23	0.25	-10.44	0.55
**AdipoQ levels- *ADIPOQ*+45T>G genotype** **(TT *vs* GG)**	-1326.5	0.43	-590.85	0.64	800.7	0.73	-	-	-	-

LON: Longevity, EAD: Early Death, P: older individuals, FL1: 1^st^ generation of longevity group, FL2: 2^nd^ generation of longevity group, FD1: 1^st^ generation of EAD Group, FD2: 2^nd^ generation of EAD Group, IGF-1: Insulin-like growth factor-1, Cholesterol ester transfer protein (CETP) TaqIB (rs708272. genotypes: B1B1, B1B2, B2B2), CETP I405V (rs5882, genotypes II, IV, VV), Angiotensin converting enzyme (ACE, rs1799752, genotype II, ID, DD), IGFBP3: IGF-binding protein 3 (IGFBP3, rs2854744, genotype AA, AC, CC) and Adiponectin (ADIPOQ, rs2241766, genotype GG, GT, TT), b-value: regression coefficient, bold highlights statistical significance with p < 0.05.

**Table 2b:** Multivariate regression analysis of gene polymorphisms and their related proteins after adjustment for age and gender.

–	**Early Death (EAD) Group**				**Longevity (LON) Group**					
–	**FD1**		**FD2**		**P**		**FL1**		**FL2**	
–	**b-value**	**p**	**b-value**	**p**	**b-value**	**p**	**b-value**	**p-Value**	**b-value**	**p**
**CETP levels- *CETP*TaqIB genotype** **(B1B1*vs* B2B2)**	-0.31	0.14	-0.34	0.10	-0.34	**0.004**	0.12	0.45	-0.62	**0.007**
**CETP levels- *CETP* I405V genotype** **(II *vs* VV)**	-0.03	0.27	-0.58	0.06	0.15	0.52	-0.11	0.71	-0.009	0.97
**ACE levels- *ACE* I/D genotype** **(II *vs* DD)**	27.59	**0.007**	18.55	**0.03**	7.89	0.43	54.22	**0.02**	26.62	**0.001**
**IGF1levels- *IGFBP3* genotype** **(AA *vs* CC)**	-3.98	0.23	-15.36	0.21	-2.94	0.62	6.49	0.32	-3.37	0.85
**AdipoQ levels- *ADIPOQ*+45T>G genotype** **(TT *vs* GG)**	-392.1	0.81	-447.68	0.65	937.5	0.73	-	-	-	-

LON: Longevity, EAD: Early Death, P: older individuals, FL1: 1^st^ generation of longevity group, FL2: 2^nd^ generation of longevity group, FD1: 1^st^ generation of EAD Group, FD2: 2^nd^ generation of EAD Group. IGF-1: Insulin-like growth factor-1, Cholesterol ester transfer protein (CETP) TaqIB (rs708272. genotypes: B1B1, B1B2, B2B2), CETP I405V (rs5882, genotypes II, IV, VV), Angiotensin converting enzyme (ACE, rs1799752, genotype II, ID, DD), IGFBP3: IGF-binding protein 3 (IGFBP3, rs2854744, genotype AA, AC, CC) and Adiponectin (ADIPOQ, rs2241766, genotype GG, GT, TT), b-value: regression coefficient, bold highlights statistical significance with p < 0.05.
