# Supplementary material for: Biomarkers and Gene Polymorphisms in Members of Long- and Short-lived Families: A Longevity Study
Source: Open Cardiovasc Med J. 2018 Jul 31;12:59–70. doi: 10.2174/1874192401812010059 (PMC6080062; doi:10.2174/1874192401812010059)
Supplement: Supplementary file 1 [file TOCMJ-12-59_SD1.pdf]

Send Orders for Reprints to reprints@benthamscience.ae

The Open Cardiovascular Medicine Journal, 2018, *12*, i-ii

BENTHAM OPEN

CrossMark

The Open Cardiovascular Medicine Journal

Supplementary Material

Content list available at: www.benthamopen.com/TOCMJ/

DOI: 10.2174/1874192401812010059

RESEARCH ARTICLE

Biomarkers and Gene Polymorphisms in Members of Long- and Short-lived Families: A Longevity Study

Vana Kolovou[1,2,*], Olga Diakoumakou[3], Athanasia K Papazafiropoulou[4], Niki Katsiki[5], Elisabeth Fragopoulou[2], Ioannis Vasiliadis[3], Dimitris Degiannis[1], Leonidas Duntas[6], Smaragdi Antonopoulou[2] and Genovefa Kolovou[3]

[1]*Molecular Immunology Laboratory, Onassis Cardiac Surgery Center, Athens, Greece*

[2]*Department of Science Nutrition-Dietetics, Harokopio University, Athens, Greece*

[3]*Cardiology Department, Onassis Cardiac Surgery Center, Athens, Greece*

[4]*1st Department of Internal Medicine and Diabetes Center, Tzaneio General Hospital of Piraeus, Greece*

[5]*2nd Propedeutic Department of Internal Medicine, Hippokration University Hospital, Thessaloniki, Greece*

[6]*Evgenideion Hospital, Unit of Endocrinology Diabetes and Metabolism, University of Athens, Athens, Greece*

Received: February 20, 2018 Revised: June 13, 2018 Accepted: June 14, 2018

SUPPLEMENTARY DATA

The PCR was performed by using KAPA Taq polymerase (KAPA Biosystems). For CETP TaqIB (rs708272) polymorphism the oligonucleotide primers, which were used are 5’-CACTAGCCCAGAGAGAGGAGTGCC-3’ (forward) and 5’-CTGAGCCCAGCCGCACACTAAC-3’ (reverse) [1]PCR product cleaved by TaqaI, creating fragments for B2 allele 535 bp and for B1 allele 361 bp and 174 bp. For CETP I405V (rs5882) polymorphism the oligonucleotide primers, which were used are 5’- TATTTTTTTCACGGATGGGCA -3’ (forward) and 5’-TTGACTGCAGGAAGCTCTGGC -3’ (reverse) [2] PCR product was cleaved by MspI, creating fragments for I allele 142 bp and for V allele 121 bp and 21bp. For ACE I/D (rs1799752) polymorphism the oligonucleotide primers, which were used are 5’-CTGGAGACCACTCCCATCCTTTCT-3’ (forward) and 5’-GATGTGGCCATCACATTCGTCAGAT-3’ (reverse) [3] The I (insertion) allele was 490bp and the D (deletion) allele was 190bp. For ADIPOQ +45T>G (rs2241766) polymorphism the oligonucleotide primers, which were used are 5’-GCAGCTCCTAGAAGTAGACTCTGCTG-3’ (forward) and 5’-GGAGGTCTGTGATGAAAGAGGCC-3’ (reverse) [4]. PCR product was cleaved by BspHI, creating fragments for T allele 206 bp and 166 bp and for G allele 372 bp. For IGFBP3 A-202C (rs2854744) polymorphism the oligonucleotide primers, which were used are 5’-CCACGAGGTACACACGAATG-3’ (forward) and 5’-AGCCGCAGTGCTCGCATCTGG-3’ (reverse) [5]. PCR product was cleaved by FspI, creating fragments for C allele 241 bp and 222 bp and for A allele 463 bp. All fragments of gene polymorphisms were subjected to electrophoresis on an agarose gel 3% and visualized with ethidium bromide.Gg

REFERENCES

[1] Fumeron F, Betoulle D, Luc G, *et al.* Alcohol intake modulates the effect of a polymorphism of the cholesteryl ester transfer protein gene on plasma high density lipoprotein and the risk of myocardial infarction. J Clin Invest 1995; 96(3): 1664-71. [http://dx.doi.org/10.1172/JCI118207] [PMID: 7657837]

[2] Gudnason V, Kakko S, Nicaud V, *et al.* Cholesteryl ester transfer protein gene effect on CETP activity and plasma high-density lipoprotein in European populations. Eur J Clin Invest 1999; 29(2): 116-28. [http://dx.doi.org/10.1046/j.1365-2362.1999.00412.x] [PMID: 10092998]

[3] Rigat B, Hubert C, Corvol P, Soubrier F. PCR detection of the insertion/deletion polymorphism of the human angiotensin converting enzyme

gene (DCP1) (dipeptidyl carboxypeptidase 1). Nucleic Acids Res 1992; 20(6): 1433.
[http://dx.doi.org/10.1093/nar/20.6.1433-a] [PMID: 1313972]

[4] Guzman-Ornelas MO, Chavarria-Avila E, Munoz-Valle JF, *et al.* Association of ADIPOQ +45T>G polymorphism with body fat mass and blood levels of soluble adiponectin and inflammation markers in a Mexican-Mestizo population. Diabetes Metab Syndr Obes 2012; 5: 369-78.
[PMID: 23118546]

[5] Deal C, Ma J, Wilkin F, *et al.* Novel promoter polymorphism in insulin-like growth factor-binding protein-3: correlation with serum levels and interaction with known regulators. J Clin Endocrinol Metab 2001; 86(3): 1274-80.
[PMID: 11238520]

© 2018 Kolovou *et al*.

This is an open access article distributed under the terms of the Creative Commons Attribution 4.0 International Public License (CC-BY 4.0), a copy of which is available at: https://creativecommons.org/licenses/by/4.0/legalcode. This license permits unrestricted use, distribution, and reproduction in any medium, provided the original author and source are credited.
